# Efficacy and safety of *Tripterygium wilfordii* polyglycosides for diabetic kidney disease: an overview of systematic reviews and meta-analyses

**DOI:** 10.1186/s13643-022-02091-3

**Published:** 2022-10-21

**Authors:** Ying Wang, Mei Han, Yao-Tan Li, Zhen Wang, Jian-Ping Liu

**Affiliations:** 1 Department of Nephrology, Dongzhimen Hospital, Beijing University of Chinese Medicine, Beijing, 100700 China; 2 Centre for Evidence-Based Chinese Medicine, Beijing University of Chinese Medicine, Chaoyang District, Beijing, 100029 China

**Keywords:** Diabetic kidney disease, *Tripterygium wilfordii* polyglycoside, Systematic review, Overview

## Abstract

**Background:**

Recently, several systematic reviews (SRs) and meta-analyses (MAs) of *Tripterygium wilfordii* polyglycoside (TWP) have reported significant benefits on diabetic kidney disease (DKD). However, the adoption of TWP for DKD remains uncommon. This study aimed to evaluate and summarize the current evidence on TWP for DKD.

**Methods:**

We searched PubMed, Web of Science, SINOMED, Embase, Cochrane Library, CNKI database, Wan Fang database, and VIP database, up to June 4, 2022. SRs of TWP on DKD were included. Two authors independently assessed eligibility, extracted data, and graded the quality of evidence. We appraised the reporting and methodological quality of the included studies based on the PRISMA statement and AMSTAR 2.

**Results:**

We included 19 SRs and MAs. Seventeen MAs of proteinuria were identified; all suggested TWP exhibited anti-proteinuria function on DKD. Of these, only 2 were graded as moderate quality of evidence. Eighteen MAs estimated the reno-protective effect of TWP; nine of them showed that TWP improved renal function, including 2 MAs rated as moderate quality of evidence. Eleven SRs showed the serum albumin level was elevated in the TWP group. Of those, four were rated as moderate quality of evidence. Fourteen MAs of the incidence of adverse events were included. Twelve MAs indicated TWP increased the risk of adverse events, of which 4 were graded with moderate quality of evidence. Twenty of the 27 items in the PRISMA checklist were adequately reported with more than 75% compliance among the included SRs, while five of the 12 items in the PRISMA checklist for abstract were found to have less than 50% compliance. The overall reporting quality of SRs published in English was higher than that in Chinese. The methodological quality of the included SRs appraised by AMSTAR-2 ranged from critically low to moderate.

**Conclusion:**

TWP appears effective for DKD on improving proteinuria and increasing the level of serum albumin, accompanied by a higher risk of adverse events. The evidence would be more credible and valuable to guide decision if the quality of the SRs and primary studies is improved.

**Systematic review registration:**

PROSPERO CRD42021249560

**Supplementary Information:**

The online version contains supplementary material available at 10.1186/s13643-022-02091-3.

## Background

Diabetic kidney disease (DKD) is CKD (chronic kidney disease) attributed to diabetes, which happens in 20–40% of patients with diabetes. DKD may occur after diabetes duration of a few years or be present at diagnosis of diabetes, along with markedly increased cardiovascular risk and healthcare costs [1]. A newly published research shows that, since 2011, the percentage of hospitalized patients in China with CKD due to diabetes has exceeded that of CKD due to glomerulonephritis, which had been the predominant cause of end-stage renal disease (ESRD) in most developing countries [2]. These pieces of evidence also indicate that the existing management approaches were insufficient to stop the progression of DKD to ESRD. New therapeutics for DKD are still urgently expected. The *Tripterygium wilfordii* polyglycoside (TWP) as a marketed Chinese patent medicine extracted from traditional Chinese medicine (TCM) *Tripterygium wilfordii* Hook F. (TwHF) has been used for treating kidney disease [3, 4]. It has also been suggested to be potentially effective for DKD through multiple pharmacological mechanism involving anti-inflammation, anti-oxidation, anti-glomerulosclerosis, and anti-fibrosis [5].

As the cornerstone of evidence-based health care, systematic reviews (SRs) and meta-analysis (MAs) have been widely adopted in various healthcare areas, including traditional Chinese medicine (TCM) [6]. Recently, there have been numerous clinical studies on the efficacy and safety of TWP on DKD, accompanied by increasing SRs and MAs on the same topic. Most of the studies have indicated that TWP exhibited benefits on reducing proteinuria of DKD. However, the certain effects of TWP on DKD, in terms of improving renal function, increasing serum albumin level, and the risk of adverse events for individuals with DKD contradicted among different SRs. This makes it difficult for health professionals to access the available information and make clinical treatment decision. As far as we know, the current evidence from different SRs about TWP on DKD has not been systematically assessed previously. We conducted this overview to compile the current evidence of the efficacy and safety of TWP for DKD from published SRs and MAs. We summarized reported outcomes and assessed the methodological quality of SRs included in this overview, as well as made suggestions for the reporting of standard outcomes in future trials and SRs. Furthermore, this overview identified gaps in the evidence base requiring further research and reviews.

## Methods

This overview of SRs and MAs is reported according to the Preferred Reporting Items for Systematic Reviews and Meta-Analyses (PRISMA) statement 2020 [7] (Additional file 1), following the recommendations of the Cochrane Collaboration Handbook [8], and was registered on the international prospective register of systematic reviews (CRD42021249560) prior to commencing this review.

### Data sources and searches

Only published SRs and MAs were considered in this overview. We searched the following databases from their inception to June 4, 2022: PubMed, Web of Science, SINOMED, Embase, Cochrane Library, China National Knowledge Infrastructure Database (CNKI), Wan Fang database, China Science and Technology Journal Database (VIP). Additionally, important conference papers were searched manually. Furthermore, the literature search was complemented by screening references in the retrieved SRs and MAs. The detail search strategy is presented in Additional file 2.

### Study selection

Two overview authors (Y. W. and Z. W.) independently reviewed the results of the research and obtained full-text version for further scrutiny. The definition of a systematic review was adopted from the Cochrane Handbook for Systematic Reviews of Interventions “A systematic review is a review of a specific research question by using an explicit and reproducible systematic methodology to identify, collect, and critically appraise all studies which meet the pre-defined eligibility criteria. Additionally, as a statistical method, meta-analysis may be used to integrate and summarize the results of the included studies, which could provide more precise estimates of the effect of health care than those derived from the individual studies included within a review” [8].

### Eligibility criteria

The eligibility criteria were checked further as follows:

1. Participants: Adults diagnosed with diabetes with CKD
2. Interventions: Treatment with TWP, without any restriction on dosage and treatment duration
3. Comparator(s)/control: There is no limitation on the pharmacological treatment in the control group (oral hypoglycemic agents, subcutaneous insulin, or placebo).
4. Outcomes: 24-h urinary protein excretion, renal function (serum creatinine, estimated glomerular filtration rate (eGFR), or creatinine clearance), serum albumin, and the incidence of adverse events
5. Types of study: Systematic reviews and meta-analyses of clinical trials regardless of randomization and blinding

Publications as protocol of SRs and MAs, narrative reviews, were excluded. Discrepancies between the two reviewers (Y. W. and Z. W.) were discussed until reaching a consensus.

### Data extraction

Two overview authors (Y. W. and Z. W.) extracted and tabulated data from included SRs and MAs independently. Extracted data were compared, with any discrepancies being resolved through discussion. We planned to contact SR authors for additional information not reported in the published version. We extracted the following data: (1) basic information of each SR (first author, year of publication, published journal, etc.); (2) types of clinical trials (RCT, controlled clinical trials, etc.); (3) details of study participants; (4) interventions and comparators; and (5) outcomes and time points.

### Methodological quality assessment

A measurement tool to assess systematic reviews 2 (AMSTAR 2) [9] was used to assess the methodological quality of included SRs and MAs and to detect the weakness in specific domains that could threaten the validity of the included SRs and MAs. There are 16 items in the checklist, each item referring to a relevant methodological aspect of the SR. Plausible scores were “no” when the SR did not meet the criteria, “yes” when the SR met the criteria, and “partial yes” when SR reported partial information on the scored item. The confidence of each SR was rated as “high,” “moderate,” “low,” or “critically low.”

### PRISMA statement adherence

The PRISMA 2009 checklist was used to score the quality of reporting by two reviewers (Y. W. and M. H.), independently. According to the explanation and elaboration document of the PRISMA [10], there are 27 items in the PRISMA checklist in total. The assessment results indicated whether each item was reported adequately or not. Certain items of the PRISMA checklist (items 14, 16, 21, and 23) might not be applicable for some studies, and they were not scored as missing nor inadequately reported in this case. Additionally, the item 2 (abstract) was scored separately according to the PRISMA for abstracts checklist [11], which contains 12 items related to details of what should be reported in the abstracts of SRs and MAs. Discrepancies were discussed until consensus was reached.

### Quality of evidence

We assessed the quality of the evidence pooled within the included SRs using GRADE (Grading of Recommendation, Assessment, Development, and Evaluation). When available, we used the GRADE assessments from the included SRs. When this was not available, we used the GRADE system to assess the following items for the quality of evidence for the main outcomes.

1. Risk of bias: Internal validity of the evidence
2. Inconsistency: Heterogeneity or variability in the estimates of effect across studies
3. Indirectness: Degree of difference between population, intervention, and outcome of interest
4. Risk of publication bias: Degree of selective publication of studies.

The GRADE system rates the quality of the evidence as follows:

1. High (further research is very unlikely to change confidence in the estimate of the effect).
2. Moderate (further research is likely to have an important impact on confidence in the estimate of effect and may change the estimate).
3. Low (further research is very likely to have an important impact on confidence in the estimate of effect and is likely to change the estimate).
4. Very low (any estimate of effect is very uncertain).

### Data analysis

The characteristics of included SRs and MAs were summarized using descriptive statistics and presented as frequency, percentage, mean with standard deviation, median with interquartile range (25 to 75% percentile) based on the type of data. We narratively summarized and presented the outcome data including effect estimates (mean difference (MD)) and 95% confidence intervals (CI) supported by statistical outcomes reported in the original SRs and MAs. For the reporting quality assessment, items of the PRISMA checklist and the PRISMA for abstract checklist were descriptively analyzed. For the methodological quality evaluation, the overall confidence of each SR was appraised and classified to four levels. We evaluated if there was a statistically significant difference between Chinese and English reviews in the quality of reporting and methodology per category, by using the *χ*^2^ test (Fisher’s exact test). The Mann-Whitney test was used to compare the overall difference in the quality of reporting SRs between Chinese and English publications. Statistical tests were performed using GraphPad Prism 9. A *p*-value of < 0.05 was considered statistically significant.

## Results

### Search and descriptive characteristics

**Fig. 1:**
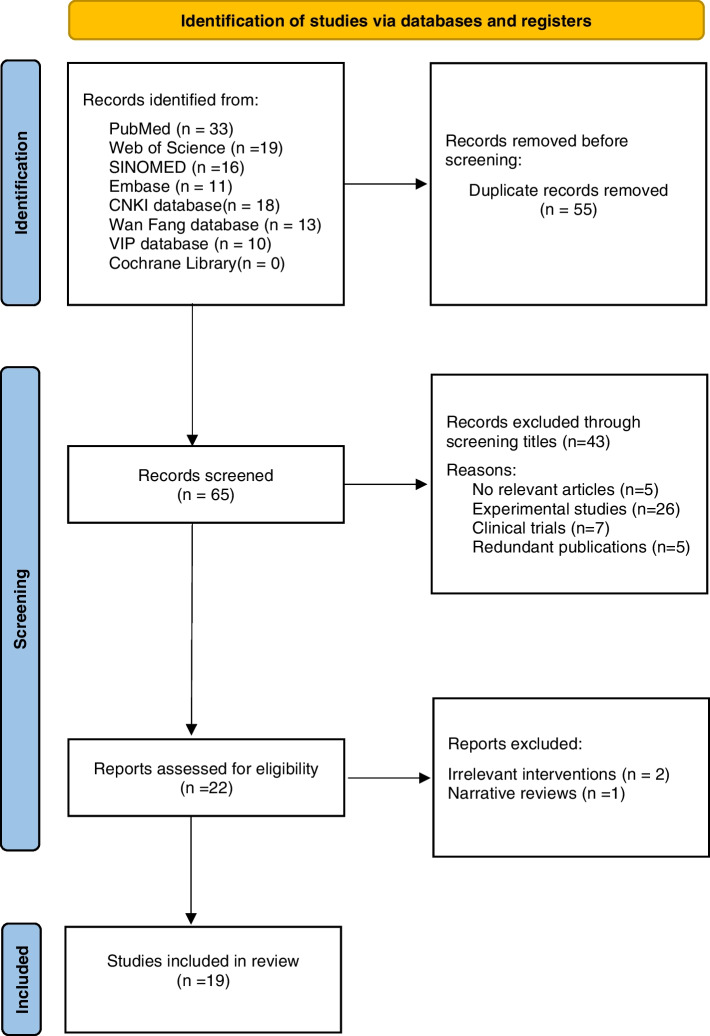
PRISMA 2020 flow diagram for new systematic reviews which included searches of databases and registers only. From: Page MJ, McKenzie JE, Bossuyt PM, Boutron I, Hoffmann TC, Mulrow CD, et al. The PRISMA 2020 statement: an updated guideline for reporting systematic reviews. BMJ 2021;372:n71. doi: 10.1136/bmj.n71. For more information, visit: http://www.prisma-statement.org/

The process of SRs and MAs identification, screening, and selection in this overview is shown in a PRISMA flow diagram (see Fig. 1). The number of records initially identified was 120. After duplications were removed, our literature search returned 65 reviews, of which 43 were excluded on the title and abstract review. Twenty-two full-text SRs were obtained for further scrutiny. Nineteen SRs and MAs were included [12–30].

We presented a table of the main characteristics of the included SRs and MAs (see Table 1). The included SRs were published between 2010 [12] and 2020 [13–18]. None of them was reported as an update of a previous SR. The SRs included a median of 14 original studies ranged from 8 [19] to 29 [20], totalling 308 clinical studies (this sum includes duplicated counts if a study is included in more than one SR). The total sample sizes for individual SRs ranged from 572 [19] to 2111 [20]. The recorded study duration ranged from 2 weeks [21] to 36 months [22]. There was overlap of included studies among the SRs. After we eliminated the duplicates, only 119 unique trials including 8626 individuals (4395 in the experimental groups and 4231 in the control groups) were actually covered by those included SRs. All the original clinical studies included in the SRs and MAs were developed and conducted in China. Six SRs were published in English, and 13 SRs were published in Chinese. Most of the SRs and MAs included RCTs of TWP treating DKD at stage 4.

**Table 1:** Characteristics of included systematic reviews and meta-analyses

Study ID	Country	Language	Stage^**a**^	***n*** (trials)	***n*** (cases)	SRs/MAs	Study type	Follow-up (months)	Main conclusion
**Wu W. H. 2010** [12]	China	Chinese	IV/NR	12	862	Both	RCT/qRCT	1~6	TWP may be a kind of medicine relatively safe and effective for DN. However, the evidence is not strong enough because of some low-quality trials and publication bias. Rigorously designed, randomized, double-blind, and placebo-controlled trials of TWP for DN are needed to further assess the effect
**Xie H. Y. 2012** [25]	China	Chinese	NR	26	1701^b^	Both	NR	NR	Due to the poor methodologic quality of the original studies, more rigorously designed, randomized, double-blind, and placebo-controlled trials of TWP on DN are needed to support the evidence
**Chen Y. 2013** [27]	China	Chinese	IV/NR	20	1414	Both	RCT/CCT	1~12	The treatment with TWP could reduce the proteinuria of DKD, but increased the risk of adverse events among DKD patients. The quality of evidence was not high
**Huang J. 2015** [28]	China	Chinese	IV	13	1119	Both	RCT/qRCT	1~6	*Tripterygium* glycosides combined with ACEI/ARB in treating diabetic nephropathy stage 4 are supper than the single administration of ACEI/ARB, with a good prospect in clinical application. Nevertheless, due to the small-size and low-quality samples in this study, more high-quality and large sample-size randomized controlled trials shall be conducted to verify the findings
**Luo J. J. 2016** [19]	China	Chinese	NR	8^b^	572^b^	Both	NR	NR	The treatment of TWP on DKD exhibited significant anti-proteinuria function, along with increased efficacy and safety, which is critically important for clinical practice
**Liang X. H. 2016** [22]	China	Chinese	NR	10	584	Both	RCT	24~36	TWP showed significantly clinical benefits on DKD. More basic experiments and large sample-size randomized controlled trials on this topic are needed
**Liao Z. M. 2016** [20]	China	Chinese	IV/NR	29	2111	Both	RCT/qRCT	1~12	*Tripterygium* glycosides treatment of diabetic nephropathy has a role in reducing proteinuria but accompanied by a decrease in plasma proteins, no significant effect on renal function
**Hong Y. 2016** [24]	China	English	IV	14	992	Both	CCT	1~6	The present evidence shows that *Tripterygium* glycosides can improve clinical efficacy, reduce the 24-h urinary protein and serum creatinine, but that they increase the Tripterygium glycoside-related toxicity in treatment of stage 4 diabetic nephropathy
**Dai X. Y. 2018** [29]	China	Chinese	IV	11	859	Both	RCT	NR	Combined use of *Tripterygium* glucosides in treating DKD stage 4 has advantages in improving clinical efficacy and decreasing ALB, but more high-quality researches are needed to make further analysis and demonstration
**Liu K. 2019** [21]	China	Chinese	NR	16	1482	Both	RCT	0.5~12	TWP combined with ACEI/ARB class drugs in the treatment of diabetic nephropathy is better than ACEI/ARB class drugs alone, with better clinical efficacy. However, due to the low quality of the included literature, high quality, large-sample randomized controlled trials are still needed for confirmation
**Zhu G. S. 2019** [26]	China	Chinese	NR	14	826	Both	RCT	1~6	The overall efficiency of TWP in the treatment of DKD is significant. It has lower UP, BUN, and Scr levels compared with RAAS blockers, while its adverse reaction incidence is comparable to that of RAAS blockers
**Ren D. J. 2019** [23]	China	English	IV	22	1414	Both	RCT	1~12	TWP combined with ARB/ACEI in the treatment of DKD stage 4 is superior to the monotherapy of ARB/ACEI
**Ye W. C. 2019** [30]	China	English	NR	12	829	Both	RCT	1~6	Combination therapy of *Tripterygium* glycosides plus valsartan may be effective for the treatment of DN. However, the safety of the combination therapy needs to be further confirmed
**Wang Y. 2020** [17]	China	English	IV	18	1160	Both	RCT	1~12	The combination treatment of TG and ARB showed promising results regarding significant proteinuria reduction and serum albumin improvement for DKD but with a higher risk of adverse events. Further higher-quality studies are necessary to provide solid evidence to determine a rational treatment strategy including TG while maximizing antiproteinuric effects and minimizing adverse events for DKD patients
**Chen H. 2020** [14]	China	Chinese	NR	13	1143	Both	RCT	3~4	Adding TWP to the routine treatment of DKD could effectively improve the patients’ body inflammation and delay the progression of DKD. Attention should be paid to abnormal liver function, leukopenia, and other adverse reactions in the treatment process
**Fang L. 2020** [15]	China	Chinese	NR	22	1736	Both	NR	1~12	TWP was weekly recommended for the treatment of DKD
**Zhang M. J. 2020** [13]	China	Chinese	NR	16	973	Both	RCT	2~6	The TWP has affirmative effect in the treatment of DKD with acceptable safety; this conclusion needs to be verified by more high-quality, large-sample, multicenter randomized double-blind controlled trials
**Fang J. Y. 2020** [18]	China	English	III~V	9	851	Both	RCT	1~12	In patients with DN, adding TGs to ACE inhibitors or ARBs significantly lowered both the 24-h UTP and SCr levels. Therefore, ACE inhibitors or ARBs plus TGs might improve the treatment of DN in patients
**Wu X. 2020** [16]	China	English	NR	23	1810	Both	RCT	0.5~12	TG combined with ARB offers a novel concept in treating DN; more high-quality RCTs are needed for better understanding and applying the combined treatment in DN

*RCT* Randomized control trial, *qRCT* Quasi-randomized control trial, *CCT* clinical control trial, *NR* not reported (there is no information provided in the full-text version of the included article), *ACEI* angiotensin-converting enzyme inhibitors/*ARB* angiotensin receptor blockers, *DN* diabetic nephropathy, *TG* tripterygium glucosides, *UP* urinary protein, *BUN* blood urea nitrogen, *Scr* serum creatinine, *ALB* blood albumin, *24-h UTP* 24-h urinary protein quantity, *Stage*^a^ the staging of DKD based on the Mogenson stage. ^b^The information was not reported directly, which was calculated according to the original publication

### Overview of the main outcomes

We summarized the main outcomes of the included SRs and MAs in Table 2. We also presented a series of tables containing information for each outcome reported from the included SRs (see Supplemental Tables 1 to 6).

**Table 2:** Summary of all outcomes within included systematic reviews and meta-analyses

Study ID	Comparison	Experimental group (TWP regimen)	Control group	Subgroups	UTP	SCR	GFR	AALB	ALT	WBC	AEs	Overall quality^**a**^
**Wu W. H. 2010** [12]	TWP+Ctrl vs Ctrl; TWP+RASi vs Ctrl	20/30/40 mg tid; 1–2 mg/kg/d; with tapering (partial studies)	Conventional therapy (or plus CTPM)	Stage of DKD	↓	ND	NR	NR	NR	NR	NR	Critically low
**Xie H. Y. 2012** [25]	TWP+Ctrl vs Ctrl	NR	Conventional therapy	No	↓	↓	NR	↑	↑	↓	NR	Critically low
**Chen Y. 2013** [27]	TWP+Ctrl vs Ctrl	10/20 mg tid; 1 mg·kg/d; 20/30/40 mg qd	Conventional therapy (or plus RASi/CTPM)	No	↓	ND	NR	↑^b^	NR	NR	↑	Critically low
**Huang J. 2015** [28]	TWP+RASi vs RASi	20 mg tid; 1 mg·kg/d; with tapering (partial studies)	RASi	Follow-up; proteinuria	↓	ND	NR	↑	NR	NR	↑	Low
**Luo J. J. 2016** [19]	NR	NR	NR	NR	↓	ND	ND	NR	NR	NR	NR	Critically low
**Liang X. H. 2016** [22]	TWP+Ctrl vs Ctrl	NR	Conventional therapy	No	↓	↓	NR	↑	NR	NR	NR	Critically low
**Liao Z. M. 2016** [20]	TWP+Ctrl vs Ctrl TWP vs ACEI	10/20/40 mg tid; 20/30/40 mg qd; 1 mg·kg/d	Conventional therapy (or plus RASi/CTPM)	No	↓	↓	NR	↑^b^	NR	NR	↑	Critically low
**Hong Y. 2016** [24]	TWP+Ctrl vs Ctrl	20 mg tid; 1 mg·kg/d	ACEI/ARB	No	↓	↓	NR	NR	NR	NR	↑	Critically low
**Dai X. Y. 2018** [29]	TWP+Ctrl vs Ctrl TWP+TCM+Ctrl vs Ctrl	NR	Conventional therapy (or plus RASi/CTPM)	No	NR	ND	NR	↑^b^	NR	NR	↑	Low
**Liu K. 2019** [21]	TWP+Ctrl vs Ctrl	20/30 mg/0.3~0.5 mg·kg tid; 1 mg·kg/d; 60–90 mg/d bid-tid	ACEI/ARB	No	↓	↓	NR	NR	NR	NR	↑	Critically low
**Zhu G. S. 2019** [26]	TWP vs Ctrl	20 mg tid; 1~2 mg·kg/d	ACEI/ARB	Follow-up	↓	ND	NR	NR	NR	NR	ND	Low
**Ren D. J. 2019** [23]	TWP+Ctrl vs Ctrl	10~40 mg tid; 20/30 mg bid; 0.5~2 mg·kg/d	ACEI/ARB	Multiple subgroups	↓	ND	NR	↑	NR	NR	↑	Critically low
**Ye W. C. 2019** [30]	TWP+Ctrl vs Ctrl	20~180 mg/d	Valsartan	Multiple subgroups	↓	ND	ND	↑	NR	NR	↑	Low
**Wang Y. 2020** [17]	TWP+Ctrl vs Ctrl	10~40 mg tid; 0.75~2 mg·kg/d; with tapering (partial studies)	ARB	Follow-up	↓	ND	ND	↑	↑	ND	↑	Moderate
**Chen H. 2020** [14]	TWP+Ctrl vs Ctrl TWP+ACEI/ARB vs Ctrl	20/40 mg tid; 1 mg·kg/d; with tapering (partial studies)	Conventional therapy (or plus RASi/CTPM)	No	NR	NR	NR	NR	↑	↓	ND	Critically low
**Fang L. 2020** [15]	TWP+Ctrl vs Ctrl TWP vs Ctrl	10~60 mg tid; 1~1.5 mg·kg/d	Conventional therapy (ACEI/ARB or CTPM)	No	↓	NR	↑	NR	NR	NR	↑	Low
**Zhang M. J. 2020** [13]	TWP+Ctrl vs Ctrl TWP vs Ctrl	10/20/40 mg tid; 20/30/40 mg qd; 0.5/1 mg·kg/d; with tapering (partial studies)	Conventional therapy (ACEI/ARB or CTPM)	Dosage regimen	↓	↓	NR	↑	ND	ND	↑	Moderate
**Fang J. Y. 2020** [18]	TWP+Ctrl vs Ctrl	40–80 mg qd; 30 mg bid; 10–20 mg tid; 0.3~0.5 mg·kg bid/tid; 1~2 mg·kg/d tid	ACEI/ARB	Follow-up	↓	↓	NR	NR	NR	NR	ND	Low
**Wu X. 2020** [16]	TWP+Ctrl vs Ctrl	10/30/40 mg tid; 0.3~0.5 mg·kg tid; 1 mg·kg/d	ARB	Follow-up	↓	↓	NR	↑	NR	NR	NR	Low

*RASi* renin angiotensin system inhibitor, *ACEI/ARB* angiotensin-converting enzyme inhibitor/angiotensin II receptor blockade, *CTPM* Chinese Traditional Patent Medicine, *Ctrl* control, *24-h UTP* 24-h urinary protein, *SCR* serum creatinine, *eGFR* estimated glomerular filtration rate, *ALB* serum albumin, *AE* adverse events, *NR* not reported. ↑Increased with significant difference; ↓decreased with significant difference; *ND* no significant difference, *overall quality*^a^ quality assessment based on AMSTAR-2 statement. ^b^The results were confirmed by the figures in the publication instead of the conclusion from the abstract

### Proteinuria

The efficacy of TWP on reducing proteinuria of individuals with DKD was investigated in 17 SRs (see Supplemental Table 1). Very low quality of evidence (7/17) to moderate quality of evidence (2/17) indicated the treatment with TWP obtained superior effect on lowering proteinuria over the control group. The pooled effect of TWP on 24-h urinary protein (UTP) ranged from −0.31 g/24 h (95% *CI*: −0.51 to −0.11) [26] to −1.85 g/24 h (95% *CI*: −2.56 to −1.14) [23], with consistent beneficial results among different subgroup analyses within the included MAs.

### Renal function

Eighteen SRs evaluated the nephroprotective role of TWP on DKD by synthesizing either serum creatinine or eGFR levels (see Supplemental Table 2). However, there was inconclusive evidence on the renal protection of TWP for DKD. Nine SRs reporting very low quality of evidence (4/9) to moderate quality of evidence (2/9) suggested a benefit by a reduction in serum creatine for participants treated with TWP, with estimate effect ranged from −0.24 μmol/L (95% *CI*: −0.40 to −0.09) [24] to −15.25 μmol/L (95% *CI*: −23.84 to −6.66) [22]. One SR providing very low quality of evidence showed a benefit on the improvement of the renal function with pooled eGFR 5.92 ml/min/1.73 m^2^ (95% *CI*: 3.14 to 8.71) [15], while eight SRs providing very low (6/8) to low (2/8) quality of evidence found no significant difference on renal function between the TWP and control groups.

### Serum albumin

Eleven SRs included serum albumin as an outcome, providing very low (2/11) to moderate (5/11) quality of evidence, showed a benefit by increasing serum albumin level with the estimate effect ranged from 0.61 g/L (95% *CI*: 0.34 to 0.87) [13] to 8.41 g/L (95% *CI*: 7.14 to 6.96) [16] (see Supplemental Table 3).

### Liver function and hematologic toxicity

Four MAs synthesized the level of alanine transaminase (ALT) and white blood-cell counts (WBC) as outcomes (see Supplemental Tables 4 and 5). Three SRs providing moderate (1/3) and low (2/3) quality of evidence demonstrated the intervention with TWP on DKD increased the ALT level [14, 17, 25], with the pooled effect ranging from 1.17 U/L (95% *CI*: 0.54 to 1.80) [17] to 3.75 U/L (95% *CI*: 2.98 to 4.53) [25]. Four SRs all showed reduction in WBC in the TWP group, although statistical significance was only reached in two of them. Those two SRs reporting low quality of evidence pooled the MD −0.22 × 10˄9/L (95% *CI*: −0.34 to −0.009) [25] and odds ratio (OR) 4.33 (95% *CI*: 1.08 to 17.47) [14].

### Adverse events

Most of the SR reviewers considered about the side effects of TWP treatment, except one [19]. Due to the unavailable incidence of adverse events offered by the original trials, only 13 of the 18 MAs assessed the potential risk of adverse events with the treatment of TWP on DKD (see Supplemental Table 6). Eleven MAs reporting low (7/11) to moderate (4/11) quality of evidence showed the incidence of adverse events was significantly higher in the TWP group than control, while two MAs reporting very low [14] and low [26] quality of evidence showed no clear difference in the incidence of adverse events between the TWP and control groups. One SR only narratively stated that there was no statistical difference between two groups [18]. Most of the adverse events reported in the SRs were associated with the side effects of TWP, including leukocyte reduction, impaired liver function, gastrointestinal reaction, and abdominal uncomfortable.

### The comparison of reporting quality between two types of SRs

The percentages of adequately reported PRISMA items and PRISMA for abstract items for the included SRs and MAs published in Chinese and English are presented in Tables 3 and 4, respectively.

**Table 3:** The comparison of each items of PRISMA adequately reported in SRs and MAs published in Chinese and English

Item	Chinese publications	English publications	***P***-value^▲^
**#1 Title**	11/13 (85%)	6/6 (100%)	*1.00*
**#2 Abstract**	12/13 (92%)	6/6 (100%)	*1.00*
**#3 Rationale**	13/13 (100%)	6/6 (100%)	NA
**#4 Objectives**	13/13 (100%)	6/6 (100%)	NA
**#5 Protocol and registration**	1/13 (8%)	1/6 (17%)	*1.00*
**#6 Eligibility criteria**	11/13 (85%)	6/6 (100%)	*1.00*
**#7 Information sources**	12/13 (92%)	6/6 (100%)	*1.00*
**#8 Search**	12/13 (92%)	6/6 (100%)	*1.00*
**#9 Study selection**	8/13 (62%)	6/6 (100%)	*0.13*
**#10 Data collection process**	11/13 (85%)	6/6 (100%)	*1.00*
**#11 Data items**	7/13 (54%)	4/6 (67%)	*1.00*
**#12 Risk of bias in individual studies**	12/13 (92%)	6/6 (100%)	*1.00*
**#13 Summary measures**	11/13 (85%)	6/6 (100%)	*1.00*
**#14 Synthesis of results**	13/13 (100%)	6/6 (100%)	NA
**#15 Risk of bias across studies**	10/13 (77%)	5/6 (83%)	1.00
**#16 Additional analyses**	8/13 (62%)	4/6 (67%)	*1.00*
**#17 Study selection**	11/13 (85%)	6/6 (100%)	*1.00*
**#18 Study characteristics**	11/13 (85%)	6/6 (100%)	*1.00*
**#19 Risk of bias within studies**	12/13 (92%)	6/6 (100%)	*1.00*
**#20 Results of individual studies**	11/13 (85%)	6/6 (100%)	*1.00*
**#21 Synthesis of results**	13/13 (100%)	6/6 (100%)	NA
**#22 Risk of bias across studies**	11/13 (85%)	6/6 (100%)	*1.00*
**#23**^a^**Additional analysis**	6/8 (75%)	5/5 (100%)	*0.49*
**#24 Summary of evidence**	12/13 (92%)	6/6 (100%)	*1.00*
**#25 Limitations**	12/13 (92%)	6/6 (100%)	*1.00*
**#26 Conclusions**	8/13 (62%)	6/6 (100%)	*0.13*
**#27 Funding**	6/13 (46%)	5/6 (83%)	*0.18*

Item 2 was assessed based on whether there was a structured abstract in the article, and the item was assessed specifically in Table 4 based on the PRISMA for abstract. ^a^Optional item, if it was done in the study and adequately reported, the item was assessed as “adequately reported”; the percentage was calculated based on the applicable studies (e.g., the item 23 was evaluated according to item 16). ^▲^Fisher’s exact test. *P*-values in italic typeface highlight a difference that was not statistically significantly different between the two journal types. *NA* not applicable

**Table 4:** The comparison of each item of PRISMA for abstract adequately reported in SRs and MAs published in Chinese and English

Item	Chinses publications	English publications	***P***-value^▲^
**1. Title**	11/13 (85%)	6/6 (100%)	*1.00*
**2. Objectives**	9/13 (69%)	6/6 (100%)	*0.26*
**3. Eligibility criteria**	5/13 (38%)	4/6 (67%)	*0.35*
**4. Information sources** (key databases searched and date of last search)	7/13 (54%)	4/6 (67%)	*1.00*
**5. Risk of bias assessment** (methods for assessing risk of bias)	2/13 (15%)	0	*NA*
**6. Included studies** (number and type of included studies and participants and relevant characteristics of studies)	8/13 (62%)	6/6 (100%)	*0.13*
**7. Synthesis of results** (results for main outcomes, preferably indicating the number of studies and participants for each. If meta-analysis was done, include summary measures and confidence intervals)	6/13 (46%)	6/6 (100%)	0.04
**8. Description of effect** (direction of the effect and size of the effect in terms meaningful to patients and clinicians)	8/13 (62%)	6/6 (100%)	*0.13*
**9. Strengths and limitations of evidence**	4/13 (31%)	1/6 (17%)	*1.00*
**10. General interpretation of the results and important implications**	9/13 (69%)	6/6 (100%)	*0.26*
**11. Funding**	0	0	NA
**12. Registration number and registry name**	0	0	NA

*P*-values in italic typeface highlight a difference that was not statistically significantly different between the two journal types

*NA* not applicable

^▲^Fisher’s exact test

The included SRs published in Chinese reported several individual items more inadequately than those published in English.

1. Registration (item 5) was reported less frequently in SRs published in Chinese (1/13) than in English (1/6).
2. One-third (2/6) of SRs published in English and 46% (6/13) of SRs in Chinese failed to list which data were sought in their studies (item 11).
3. One-third (2/6) of SRs published in English and 38% (5/13) of SRs in Chinses did not describe the methods of additional analyses in their studies (item 16).
4. A total of 54% (7/13) of the SRs published in Chinese and 17% (1/6) of English SRs described neither the sources of funding nor the role of funders for the studies (item 27).
5. A total of 48% (5/13) of the SRs published in Chinese stated neither the process for selecting studies (item 9) nor a general interpretation of the results in the context of other evidence (item 26), whereas both items (items 9 and 26) were adequately reported in SRs published in English.

In sum, the SRs published in Chinese and English reported a median of 85% and 100% of PRISMA items, respectively. The overall difference in the adequate reporting of PRISMA items between the two types of SRs is statistically significant (*p* < 0.01).

In contrast to the assessment of main text, SRs included in this overview reported items of the PRISMA for abstracts checklist unsatisfactorily. The included SRs reported several individual items of PRISMA for abstract improperly in both types of publications. Two items were consistently ignored by all reviewers. None of them provided information on the main source of funding (item 11) and registration (item 12) in the abstract. The included SRs published in English reported most of the items adequately more frequently than in Chinese, except two items (items 5 and 9 of the PRISMA for abstract). None of the included SRs published in English described any method that was used to assess the risk of bias in the original trials in the abstract section, whereas 15% (2/13) of the included Chinese SRs reported this item. Only 17% (1/6) of the included SRs published in English summarized strengths and limitations of evidence in the abstract, while 31% (4/13) of the SRs published in Chinese stressed that item. A total of 54% (7/13) of the SRs published in Chinese did not report the results of main outcomes in the abstract (item 7 of the PRISMA for the abstract), while all SRs published in English presented the main outcomes in the abstract adequately. In sum, the SRs published in Chinese and English reported a median of 50% and 83% of PRISMA items for abstract, respectively. There is no significant difference in the reporting of PRISMA items for abstract between the two types of SRs.

### The comparison of methodological quality between two types of SRs

The methodological quality was assessed as critically low (10/19), low (7/19), and moderate (2/19), by using AMSTAR 2 (Supplemental Table 7). As for individual domains evaluated, all SRs had shortcomings in providing the sources of funding for the original trials (item 10). Only 11% (2/19) of the SRs provided registration information. Additionally, there was no extra information about the adjustment from the pre-specified methods in the included SRs (item 2). Only 11% (2/19) of the SRs authors assessed the potential impact of risk of bias in original individual studies on the results of the meta-analyses (item 12). A total of 42% (8/19) of the included SRs failed to provide either satisfactory explanation and discussion of any heterogeneity existed in the results of the SRs (item 14) or a potential source of conflict of interest (item 16). Although most of the domains of the AMSTAR 2 were adequately performed (with higher percentage) in the studies published in English than in Chinese, the overall confidence in the results of the SRs was apprized similarly between the two types of publication (see Table 5).

**Table 5:** The comparison of methodological quality between Chinese and English SRs

	Critically low	Low	Moderate	High
**Chinese publications**	8/13 (62%)	4/13 (31%)	1/13 (8%)	0
**English publications**	2/6 (33%)	3/6 (50%)	1/6 (17%)	0
**Z**	−1.124			
***P*-values**^▲^	*0.261*			

*P*-values in italic typeface highlight a difference that was not statistically significantly different between the two journal types

^▲^Mann-Whitney test

### Evidence quality of outcomes

The results of evidence quality rated by GRADE were very low (30.2%, 16/53), low (45.3%, 24/53), and moderate (24.5%, 13/53) (see Supplemental Table 8). Risk of bias was the most common item for downgrading the quality of the evidence due to the unclear information about the “allocation concealment” and “blinding of participants and personnel.” No evidence was downgraded because of indirectness.

## Discussion

### Summary of main findings and implications for clinicians and future study

This overview systematically reviewed the efficacy and safety of TWP for DKD by summarizing currently available evidence from SRs and MAs. The accumulated evidence for TWP showed that it could provide benefits on reducing proteinuria and elevating serum albumin, along with increased risk of adverse events for DKD. The commonly assessed outcomes were the level of ALT and WBC in terms of adverse events associated with the intervention with TWP. Clinicians should be more alerted to the adverse effects caused by the TWP. Both the liver function and the WBC counts should be carefully monitored during the administration of TWP. There is inconsistent evidence on the renal protective effect of TWP for DKD, as referred to the level of serum creatinine and eGFR. In addition, there was no high quality of evidence on the efficacy of TWP for DKD. Thereby, higher quality of evidence about the potential benefits of TWP on DKD is warranted to draw a conclusion.

We assessed the quality of SRs reporting, based on the PRISMA statement and checklists for both the full text and abstract, respectively. The results showed that the current reporting quality of the SRs and MAs on the efficacy and safety of TWP on DKD was suboptimal. We identified a few aspects inadequately reported, in which improvement is clearly needed (1). Authors should provide registration information for the SR, otherwise state the SR was not registered. The registration and pre-specified protocol can facilitate the transparency and reproducibility of the SRs, avoid repetitive efforts, minimize potential bias in the conducting and reporting of the SRs, and promote updating of SRs [31, 32] (2). SR authors should list and define all data items they sought, even if the information was not available. Furthermore, reviewers should state if there is any deviation of the methods to those already pre-specified before the SR started. It is also encouraged to describe any assumption made about any missing or unclear information from the original trials (3). It is important to disclose any financial support the SR authors received to conduct the SR or other accesses to databases that would otherwise not be available to the authors. Additionally, the essential element that was barely declared was the role of the sponsors in the SRs. Even if there is no financial support for the study, authors should state explicitly the review was not funded. To improve the transparency of the financial support and avoid any possible conflicts of interest, SR authors can use the checklist developed by the International Committee of Medical Journal Editors (ICMJE).

An abstract presented the most critical information about the SRs, which helps readers getting an awareness of the question the review addressed and deciding whether to access the full article. In some cases, the abstract may be all that most readers have access to [7]. The overall adherence to the PRISMA for abstract of the English publications was much higher than those published in Chinese, especially in the term of describing the synthesis of results in the “Results” section of abstract. Even given space limitations, the results for the main outcomes should be presented in the abstract, with the summary measure and confidence interval (if meta-analyses have been conducted). In certain cases (e.g., when the proportion of the total number of participants that contributed to a particular outcome was small), the number of studies and participants should be stated as well [11].

None of the included SRs was rated as high in confidence in the results. One common issue was that almost all SR authors failed to state that the review methods had been established ahead of conducting the review. Even if there was a registered protocol, the discrepancies between the published SRs and the planned protocol were unexplained. One more critical item, which needs more attention, is the potential impact of risk of bias in individual studies on meta-analyses results was inadequately examined. Since most of the clinical trials included in the SRs had variable quality, reviewers should take account of the impact of risk of bias in the study level on the results of the SRs [33].

### Recommendation

Further higher-quality SRs are necessary to provide solid evidence on the renal protective function of TWP for DKD. SR authors should develop a protocol and register for the SRs and (/or) MAs prior to conducting the reviews according to the PRISMA-P statement. It is also necessary for reviewers to report their outcomes according to the PRISMA statement. Although the poor quality of the original studies limited the reliability of SRs, the methodological quality of the SRs needs to be improved by SR reviewers. Even more to the point, as reported in most of the published SRs, the original clinical trials were poorly conducted, so the fervent desire to systematically review the RCTs should divert to the improvement of the fundamental clinical trials first.

### Strengths and limitation

To the best of our knowledge, this is the first overview of available SRs concerning the efficacy and safety of TWP for DKD. This overview offered a summary of the current evidence based on the published SRs and MAs, as well as critical appraisal of included SRs according to generally acknowledged tools. Indeed, there were a few limitations of this overview:

1. Although we used less restrictive search strategies to retrieve more potentially relevant articles, there were articles with neither formal titles nor appropriate keywords, which could not be retrieved.
2. All of the original clinical trials enrolled were conducted in China. No evidence from other populations than Chinese was available, which restricted the applicability of the conclusion of this overview.
3. As an overview, the summarized outcomes presented in the results did not provide information on the magnitude of effects nor account for differences in the relative sizes of the SRs.
4. The very low to moderate quality of evidence, the fair reporting quality, and the critically low to moderate methodological quality of the included SRs affected the solidity of the conclusion.

## Conclusion

Very low to moderate quality of evidence indicated that the extra administration of TWP exhibited a superior effect on reducing proteinuria and improving serum albumin, but accompanied by a higher risk of adverse events, compared with conventional therapy (interventions without TWP) for DKD. Health providers should balance the potential benefits of treatment against uncertainty related to the evidence and the underlying side effects before prescribing TWP to DKD patients. Overall, the published SRs of TWP on DKD were evaluated as fair reporting quality and inadequate methodological quality. SR authors should adhere to the AMSTAR and PRISMA statements to improve the quality of SRs in this field.

## Supplementary Information

**Additional file 1.** PRISMA Checklist.

**Additional file 2.** Search Strategy for English Database.

**Additional file 3: Supplemental Table 1.** Overview of 24-hour Urinary Protein in the Included SRs and MAs. **Supplemental Table 2.** Overview of the Included SRs and MAs of Renal Function. **Supplemental Table 3.** Overview of the Included SRs and MAs about the Outcome of Serum Albumin. **Supplemental Table 4.** Overview of the Included SRs and MAs of AL. **Supplemental Table 5.** Overview of the included SRs and MAs about the outcomes of WBC. **Supplemental Table 6.** Overview of the Incidence of Adverse Events in the Included SRs and MAs. **Supplemental Table 7.** Methodological Quality Assessment of the Systematic Reviews and Meta-analyses Based on AMSTAR-2 tool. **Supplemental Table 8.** Quality of Evidence in Included SRs with GRADE.

## Data Availability

All data generated or synthesized during this study are included in this published article and supplementary material.

## Abbreviations

TWP: *Tripterygium wilfordii* polyglycoside
TCM: Traditional Chinese medicine
DKD: Diabetic kidney disease
SR: Systematic review
MA: Meta-analysis
CNKI: China National Knowledge Infrastructure Database
CBM: Chinese Biomedical Literature Database
DN: Diabetic nephropathy
AMSTAR: A measurement tool to assess systematic reviews
PRISMA: Preferred Reporting Items for Systematic reviews and Meta-Analyses
