# Supplementary material for: Efficacy and safety of Tripterygium wilfordii polyglycosides for diabetic kidney disease: an overview of systematic reviews and meta-analyses
Source: Syst Rev. 2022 Oct 21;11:226. doi: 10.1186/s13643-022-02091-3 (PMC9585776; doi:10.1186/s13643-022-02091-3)
Supplement: Supplementary file 2 — Additional file 2. Search Strategy for English Database. [file 13643_2022_2091_MOESM2_ESM.pdf]

Supplemental file 2. Search strategy

Database	Methods（Filters）	Search term			Searching strategy	Results
		#1	#2	#3		
PubMed	[Title/Absract]	“Tripterygium Glycosides” or “Tripterygium wilfordii” or “lei gong teng” or “thunder god vine”	“Diabetic kidney disease” or “Diabetic nephropathy” or “DKD” or “DN”	“systematic review” or “meta-analysis” or “SR” or “MA”	#1+#2+#3	33
Web of Science	topic					19
Embase	Title/Abstract/Keyword					11
SinoMed	Common field					16
CNKI	Title or Abstract					18
WanFang	Title or Abstract					13
VIP	Title or Abstract					10
Cochrane Library	Title,abstract,keyword					0
