# Supplementary material for: Efficacy and safety of Tripterygium wilfordii polyglycosides for diabetic kidney disease: an overview of systematic reviews and meta-analyses
Source: Syst Rev. 2022 Oct 21;11:226. doi: 10.1186/s13643-022-02091-3 (PMC9585776; doi:10.1186/s13643-022-02091-3)
Supplement: Supplementary file 3 — Additional file 3: Supplemental Table 1. Overview of 24-hour Urinary Protein in the Included SRs and MAs. Supplemental Table 2. Overview of the Included SRs and MAs of Renal Function. Supplemental Table 3. Overview of the Included SRs and MAs about the Outcome of Serum Albumin. Supplemental Table 4. Overview of the Included SRs and MAs of AL. Supplemental Table 5. Overview of the included SRs and MAs about the outcomes of WBC. Supplemental Table 6. Overview of the Incidence of Adverse Events in the Included SRs and MAs. Supplemental Table 7. Methodological Quality Assessment of the Systematic Reviews and Meta-analyses Based on AMSTAR-2 tool. Supplemental Table 8. Quality of Evidence in Included SRs with GRADE. [file 13643_2022_2091_MOESM3_ESM.zip › 13643_2022_2091_MOESM3_ESM/ST 7_AMSTAR 2 (20221013)_ESM.pdf]

Supplemental Table 7. Methodological Quality Assessment of the Systematic Reviews and Meta-analyses Based on AMSTAR-2 tool

Study ID	1	2★	3	4★	5	6	7★	8	9★	10	11★	12	13★	14	15★	16	Total yes n(%)	Overall quality
Wu WH 2010	✓	✗	✓	✓	✓	✓	✗	✓	?	✗	✓	✗	✓	✓	✓	✓	11(68.75)	**CL**
Xie HY 2012	✓	✗	✗	✓	✗	✗	✗	✗	✓	✗	✓	✗	✓	✓	✗	✗	6(37.50)	**CL**
Chen Y 2013	✓	✗	✓	✓	✗	✓	✗	✗	✓	✗	✓	✓	✓	✗	✓	✗	9(56.25)	**CL**
Huang J 2015	✓	✗	✓	✓	✓	✓	✓	✓	✓	✗	✓	✓	✓	✓	✓	✓	14(87.50)	**L**
Luo JJ 2016	✓	✗	✗	✗	✗	✗	✗	✗	✗	✗	✗	✗	✗	✗	✗	✗	1(6.25)	**CL**
Liang XH 2016	✓	✗	✓	✓	✓	✓	✓	?	✓	✗	?	✗	✓	✓	✓	✗	10(62.50)	**CL**
Liao ZM 2016	✓	✗	✓	✓	✓	✓	✗	?	✓	✗	✓	✗	✓	✗	✓	✗	9(56.25)	**CL**
Hong Y 2016	✓	✗	✓	✓	✗	✓	✓	✓	✓	✗	✓	✗	✗	✗	✓	✓	10(62.50)	**CL**
Dai XY 2018	✓	✗	✓	✓	✓	✗	✓	✗	✓	✗	✓	✗	✓	✗	✓	✓	10(62.50)	**L**
Liu K 2019	✓	✗	✓	✓	✓	✓	✗	✓	✓	✗	✓	✗	✓	✗	✓	✗	10(62.50)	**CL**
Zhu GS 2019	✓	✗	✓	✓	✓	✓	✓	✓	✓	✗	✓	✗	✓	✓	✓	✓	13(81.25)	**L**
Ren D.J 2019	✓	✗	✓	✓	✓	✓	✓	✓	✓	✗	✓	✗	✗	✓	✓	✓	12(75.00)	**CL**
Ye W.C 2019	✓	✗	✓	✓	✓	✓	✓	?	✓	✗	✓	✗	✓	✓	✓	✓	12(75.00)	**L**
Wang Y 2020	✓	✓	✓	✓	✓	✓	✓	✓	✓	✗	✓	✗	✓	✓	✓	✓	14(87.50)	**M**
Chen H 2020	✓	✗	✓	✓	✓	✓	✓	✓	✓	✗	✓	✗	✗	✓	✓	✗	11(68.75)	**CL**
Liu F 2020	✓	✗	✓	✓	✓	✓	✓	✓	✓	✗	✓	✗	✓	✓	✓	✗	12(75.00)	**L**
Zhang MJ 2020	✓	✓	✓	✓	✓	✓	✓	✓	✓	✗	✓	✗	✓	✗	✓	✓	13(81.25)	**M**
Fang J.Y 2020	✓	✗	✓	✓	✓	✓	✓	✓	✓	✗	✓	✗	✓	✗	✓	✓	12(75.00)	**L**
Wu X 2020	✓	✗	✓	✓	✓	✓	✓	✓	✓	✗	✓	✗	✓	✓	✓	✓	13(81.25)	**L**
In total of "Y" (%)	100	10.53	89.47	94.74	78.95	84.21	68.42	63.16	89.47	0	89.47	10.53	78.95	57.89	89.47	57.89		

★=critical domains in AMSTAR 2; "✓"="Yes"; "?"="Partial Yes"; "✗"="No"; H=high, M=moderate, L=low, CL= Critically low.

Rating overall confidence in the results of the SR (shttps://www.bmj.com/content/358/bmj.j4008)

• High= No or one non-critical weakness: the systematic review provides an accurate and comprehensive summary of the results of the available studies that address the question of interest

• Moderate=More than one non-critical weakness*: the systematic review has more than one weakness but no critical flaws. It may provide an accurate summary of the results of the available studies that were included in the review

• Low=One critical flaw with or without non-critical weaknesses: the review has a critical flaw and may not provide an accurate and comprehensive summary of the available studies that address the question of interest

• Critically low=More than one critical flaw with or without non-critical weaknesses: the review has more than one critical flaw and should not be relied
