# Supplementary material for: Efficacy and safety of Tripterygium wilfordii polyglycosides for diabetic kidney disease: an overview of systematic reviews and meta-analyses
Source: Syst Rev. 2022 Oct 21;11:226. doi: 10.1186/s13643-022-02091-3 (PMC9585776; doi:10.1186/s13643-022-02091-3)
Supplement: Supplementary file 3 — Additional file 3: Supplemental Table 1. Overview of 24-hour Urinary Protein in the Included SRs and MAs. Supplemental Table 2. Overview of the Included SRs and MAs of Renal Function. Supplemental Table 3. Overview of the Included SRs and MAs about the Outcome of Serum Albumin. Supplemental Table 4. Overview of the Included SRs and MAs of AL. Supplemental Table 5. Overview of the included SRs and MAs about the outcomes of WBC. Supplemental Table 6. Overview of the Incidence of Adverse Events in the Included SRs and MAs. Supplemental Table 7. Methodological Quality Assessment of the Systematic Reviews and Meta-analyses Based on AMSTAR-2 tool. Supplemental Table 8. Quality of Evidence in Included SRs with GRADE. [file 13643_2022_2091_MOESM3_ESM.zip › 13643_2022_2091_MOESM3_ESM/ST-1_UTP 2021-06-21R2.pdf]

Supplemental Table 1. Overview of 24-hour Urinary Protein in the Included SRs and MAs

Study ID (first author year)	N (studies)	follow-up (months)	N (cases)	comparison	subgroups	I^2(%)	MD/SMD (g/24h)	95%CI [,]	P	Certainty
Wu W.H. 2010	4	2～6	389	TWP+CT vs CT (5); TWP+ACEI/ARB vs Ctrl (6)	clinical stage	57	-0.49	-0.63,-0.34	< 0.00001	Very low
	7	1～6	457		without staging	83	-0.6	-0.96,-0.24	=0.001	Very low
Xie H.Y. 2012	22	NR	NR	TWP+Ctrl vs Ctrl	no	NR	-0.66	-0.72,-0.59	< 0.00001	Low
Chen Y 2013	20	NR	1414	TWP+CT vs CT (15); TWP +ACEI/ARB vs ACEI/ARB (5); TWP+CTPM vs CTPM (3)	no	81	-0.61	-0.76,-0.45	< 0.00001	Very low
Huang J 2015	12	1～6	1033	TWP+ACEI/ARB vs ACEI/ARB	no	89	-0.84	-1.02,0.66	< 0.00001	Low
Hong Y 2016	13	1 ~ 6	NR	TWP+ACEI/ARB vs ACEI/ARB	no	91.1	-1.55	-2.06,-1.03	< 0.00001	Low
Luo J.J. 2016	4	NR	381	NR	no	NR	-0.48	-0.62,-0.33	< 0.05	Very low
Liang X.H. 2016	6	24/36	275	TWP+CT vs CT	no	80	-0.66	-1.05,-0.23	< 0.05	Low
Liao Z.M. 2016	29	1～12	2121	TWP+CT vs CT (11); TWP+ACEI/ARB vs ACEI/ARB (10); TWP+CTPM vs CTPM (7); TWP vs ACEI (1)	no	94	-0.85	-1.11,-0.59	< 0.00001	Very low
Ren D.J. 2019	21	1 ~ 6	1595	TWP (+ACEI/ARB) vs ACEI/ARB	total effect	87	-0.87	-1.03,-0.71	NR	Low
	8		478		follow-up period < 3months	89	-1.85	-2.56,-1.14	< 0.00001	
	8		433		follow-up period 3～6months	88	-1.39	-2.03,-0.76	< 0.0001	
	10		684		follow-up period ≥6months	77	-1.37	-1.73,-1.01	< 0.0001	
	5		317		UTP≥1.0g/d	84	-0.63	-1.00,-0.25	< 0.0001	
	8		537		UTP≥1.5g/d	92	-0.72	-0.97,-0.47	< 0.0001	
	9		476		UTP≥3.5g/d	42	-1.10	-1.26,-0.94	< 0.0001	
Ye W.C. 2019	12	1 ~ 6	829	TWP + valsartan vs valsartan	no	90	-0.97	-1.19,-0.76	< 0.0001	Moderate
	7		NR		publication year ≤ 2013	87.2	-1.02	-1.28,-0.77	< 0.01	
	5				publication year > 2013	89.6	-0.90	-1.19,-0.76		

	8				sample size ≤70	86	-0.97	-1.24,-0.69		
	4				sample size > 70	90	-0.98	-1.37,-0.6		
	4				valsartan dosages ≤80mg/d	89.3	-0.90	-1.47,-0.33		
	8				valsartan dosages > 80mg/d	91.0	-1.00	-1.25,-0.75		
	9				TWP dosages ≤60mg/d	91.8	-0.94	-1.18,-0.71		
	3				TWP > dosages 60mg/d	73.5	-1.12	-1.74,-0.49		
	6				follow-up period ≤3months	90.2	-0.88	-1.14,-0.62		
	6				follow-up period > 3months	90.1	-1.05	-1.50,-0.61		
Liu K 2019	16	0.5 ~ 12	1475	TWP+ACEI/ARB vs ACEI/ARB	no	97	-1.27	-2.04,-0.51	0.001	Very low
Zhu G.S. 2019	9	1 ~ 6	498	TWP vs ACEI/ARB	total effect	74	-0.53	-0.77,-0.29	< 0.0001	Low
	3	1/2	180		follow-up period < 3months	0	-1.00	-1.25,-0.76	< 0.00001	
	3	3	154		follow-up period =3months	43	-0.31	-0.51,-0.11	0.002	
	3	6	164		follow-up period > months	57	-0.49	-0.92,-0.06	0.02	
Wang Y 2020	17	1 ~ 12	1033	TWP+ARB vs ARB	total effect	79	-0.95	-1.17,-0.74	< 0.00001	Low
	7		296		< 6months	8	-0.57	-0.71,-0.42	< 0.00001	
	10		737		≥6months	61	-1.24	-1.46,-1.02	< 0.00001	
Fang J.Y. 2020	9	1 ~ 12	851	TWP+ACEI/ARB vs ACEI/ARB	total effect	79	-0.34	-0.38,-0.30	< 0.00001	Moderate
	1		216		follow-up period < 2months	NA	-0.25	-0.32,-0.18	NA	
	7		495		follow-up period 2～6months	43	-0.39	-0.44,-0.33	< 0.00001	
	1		140		follow-up period > 6months	NA	-2.09	-2.89,-1.29	NA	
Wu X 2020	18	0.5 ~ 12	1339	TWP+ARB vs ARB	total effect	89	-1.46	-1.84,-1.09	< 0.00001	Very low
	8		621		follow-up period ≤14weeks	88	-1.32	-1.86,-0.78	< 0.00001	
	10		718		follow-up period > 14weeks	88	-1.57	-2.09,-1.06	< 0.00001	
Zhang M.J. 2020	16	2 ~ 6	1015	TWP vs CT (7); TWP + ARB vs ARB (6); TWP vs ARB (1)	no	71	-0.78	-1.03,-0.54	< 0.00001	Low

				TWP + CTPM vs CTPM (2); comparison with different dose (1)						
Liu F 2020	16	1 ~ 12	1146	TWP+CT vs CT (1); TWP+ARB vs ARB (7); TWP+ACEI vs ACEI (4); TWP+ACEI/ARB vs ACEI/ARB (1); TWP+other agents vs other agents (3)	no	76	-1.01	-1.27,-0.75	< 0.00001	Very low

Notes: NR=Not Reported (there is no information provided in the full text version of the included article); NA=Not Applicable; ACEI/ARB = angiotensin-converting enzyme inhibitor/angiotensin II receptor blockade; CTPM=Chinese Traditional Patent Medicine; Ctrl=control; UTP =24hour urinary protein.
