# Supplementary material for: Efficacy and safety of Tripterygium wilfordii polyglycosides for diabetic kidney disease: an overview of systematic reviews and meta-analyses
Source: Syst Rev. 2022 Oct 21;11:226. doi: 10.1186/s13643-022-02091-3 (PMC9585776; doi:10.1186/s13643-022-02091-3)
Supplement: Supplementary file 3 — Additional file 3: Supplemental Table 1. Overview of 24-hour Urinary Protein in the Included SRs and MAs. Supplemental Table 2. Overview of the Included SRs and MAs of Renal Function. Supplemental Table 3. Overview of the Included SRs and MAs about the Outcome of Serum Albumin. Supplemental Table 4. Overview of the Included SRs and MAs of AL. Supplemental Table 5. Overview of the included SRs and MAs about the outcomes of WBC. Supplemental Table 6. Overview of the Incidence of Adverse Events in the Included SRs and MAs. Supplemental Table 7. Methodological Quality Assessment of the Systematic Reviews and Meta-analyses Based on AMSTAR-2 tool. Supplemental Table 8. Quality of Evidence in Included SRs with GRADE. [file 13643_2022_2091_MOESM3_ESM.zip › 13643_2022_2091_MOESM3_ESM/ST-2_Scr 20210621R2.pdf]

Supplemental Table 2. Overview of the Included SRs and MAs of Renal Function

Study ID	N (studies)	follow-up period (months)	N (cases)	Comparison	Subgroups	I^2(%)	MD/SMD (μmol/L)	95%CI [,]	P	Certainty
Wu W.H 2010	4	2～6	369	TWP+Ctrl vs Ctrl	clinical stage	88	-8.43	-18.15,1.29	0.09	Very low
	7	1～6	513	TWP+ACEI/ARB vs Ctrl	without staging	39	-0.66	-2.12,0.79	0.37	
Xie H.Y 2012	21	NR	NR	TWP+Ctrl vs Ctrl	no	NR	-15.16	-16.14,14.18	＜0.00001	Low
Chen Y 2013	16	NR	1159	TWP+CT vs CT<br>TWP +ACEI/ARB vs ACEI/ARB<br>TWP+CTPM vs CTPM	no	96	-1.68	-7.46,4.09	0.57	Very low
Huang J 2015	10	1～6	787	TWP +ACEI/ARB vs ACEI/ARB	total effect	85	-0.82	-4.30,2.66	0.64	Low
	2		146		24UTP＞0.5g	0	-3.06	-6.35,0.23	0.07	
	2		149		24UTP＞1.0g	0	-14.52	-18.13,10.91	＜0.00001	
	2		142		24UTP＞1.5g	35	0.74	-2.02,3.50	0.60	
	3		360		24UTP＞3.5g	0	3.04	1.17,4.90	0.001	
Hong Y2016	10	1 ~ 6	NR	TWP +ACEI/ARB vs ACEI/ARB	no	82.3	-0.24	-0.40,-0.09	＜0.05	Low
Luo J.J 2016	4	NR	361	NR	no	NR	-8.32	-17.04,1.18	＞0.05	Very low
Liang X.H 2016	7	24/36	446	TWP+CT vs CT	no	95	-15.25	-23.84,-6.66	0.0005	Moderate
Liao Z.M 2016	24	1 ~ 12	1771	TWP+CT vs CT<br>TWP+ACEI/ARB vs ACEI/ARB<br>TWP+CTPM vs CTPM<br>TWP vs ACEI	no	90	-4.35	-5.14,-3.56	＜0.00001	Very low
Dai X.Y 2018	2	NR	NR	TWP+ACEI/ARB+TCPM vs ACEI/ARB+TCPM	no	86	-6.78	-19.84,6.28	0.31	Very low
Ren D.J 2019	18	1 ~ 6	NR	TWP (+ACEI/ARB) vs ACEI/ARB	no	83	-3.02	-6.40,0.37	＞0.05	Very low
Ye W.C 2019	9	1 ~ 6	532	TWP+ valsartan vs valsartan	total effect	84	-0.26	-7.52,7.00	0.94	Low

	6				publication year≤2013	89.6	0.22	-8.88,9.33	0.962	
	3				publication year > 2013	0	-1.80	-10.99,7.40	0.702	
	6				sample size ≤70	25.1	2.27	-1.59,6.12	0.249	
	3				sample size > 70	59.6	-6.18	-19.25,2.88	0.354	
	3		NR		valsartan dosages≤80mg/d	0	1.37	-2.53,5.26	0.491	
	6				valsartan dosages > 80mg/d	87.8	0.11	-11.05,11.27	0.984	
	7				TWP dosages≤60mg/d	87.7	0.79	-7.40,8.97	0.851	
	2				TWP > dosages 60mg/d	0	-5.32	-17.06,6.43	0.375	
	3				follow-up period≤3months	8.5	2.29	-3.18,7.76	0.412	
	6				follow-up period > 3months	86.9	0.27	-9.45,9.99	0.957	
Liu K 2019	10	2 ~ 12	887	TWP+ACEI/ARB vs ACEI/ARB	no	76	-7.49	-13.83,-1.15	0.02	Very low
Zhu G.S 2019	4	1 ~ 6	196	TWP vs ACEI/ARB	no	0	-5.47	-11.04,0.11	0.05	Low
Wang Y2020	14	1 ~ 12	904	TWP+ARB vs ARB	total effect	76	-1.58	-7.12,3.95	0.57	Very low
	6		333		follow-up period < 6months	0	2.14	-1.47,5.74	0.25	
	8		571		follow-up period≥6months	82	-3.19	-12.04,5.66	0.48	
Fang J.Y 2020	8	1 ~ 12	781	TWP+ACEI/ARB vs ACEI/ARB	no	19	-9.87	-13.76,-5.97	< 0.00001	Moderate
Wu X 2020	18	0.5 ~ 12	1397	TWP+ARB vs ARB	total effect	93	-7.65	-12.99,-2.31	0.005	Very low
	10				follow-up period ≤14weeks	67	-3.10	-7.41,1.21	0.16	
	8				follow-up period > 14weeks	95	-11.14	-19.45,-2.84	0.009	
Liu F 2020	2	1 ~ 12	269	TWP+Ctrl vs Ctrl; TWP vs Ctrl	no	46	5.92	3.14,8.71	< 0.0001	Very low
Zhang M.J 2020	15	2 ~ 6	950	TWP vs CT<br>TWO + ARB vs ARB<br>TWP vs ARB<br>TWP + CTPM vs CTPM<br>comparison with different dose	no	89	-0.44	-0.85,-0.03	0.04	Very low

Notes: NR=Not Reported (there is no information provided in the full text version of the included article); NA=Not Applicable; ACEI/ARB = angiotensin-converting enzyme inhibitor/angiotensin II receptor blockade; CTPM=Chinese Traditional Patent Medicine; Ctrl=control; UTP =24hour urinary protein.
