# Supplementary material for: Efficacy and safety of Tripterygium wilfordii polyglycosides for diabetic kidney disease: an overview of systematic reviews and meta-analyses
Source: Syst Rev. 2022 Oct 21;11:226. doi: 10.1186/s13643-022-02091-3 (PMC9585776; doi:10.1186/s13643-022-02091-3)
Supplement: Supplementary file 3 — Additional file 3: Supplemental Table 1. Overview of 24-hour Urinary Protein in the Included SRs and MAs. Supplemental Table 2. Overview of the Included SRs and MAs of Renal Function. Supplemental Table 3. Overview of the Included SRs and MAs about the Outcome of Serum Albumin. Supplemental Table 4. Overview of the Included SRs and MAs of AL. Supplemental Table 5. Overview of the included SRs and MAs about the outcomes of WBC. Supplemental Table 6. Overview of the Incidence of Adverse Events in the Included SRs and MAs. Supplemental Table 7. Methodological Quality Assessment of the Systematic Reviews and Meta-analyses Based on AMSTAR-2 tool. Supplemental Table 8. Quality of Evidence in Included SRs with GRADE. [file 13643_2022_2091_MOESM3_ESM.zip › 13643_2022_2091_MOESM3_ESM/ST-3_serum albuminR2.pdf]

Supplemental Table 3. Overview of the Included SRs and MAs about the Outcome of Serum Albumin

Study ID	N (studies)	follow-up period (months)	N (cases)	comparison	subgroups	I^2(%)	MD (g/L)	95%CI [,]	P	certainty
Xie H.Y 2012	15	NR	NR	TWP+Ctrl vs Ctrl	no	NR	1.54	0.95,2.72	< 0.00001	low
Chen Y 2013	11	NR	652	TWP+CT vsCT<br>TWP +ACEI/ARB vs ACEI/ARB<br>TWP+CTPM vs CTPM	no	85	2.04	0.42,3.66	0.01	Low
Huang J 2015	9	1～6	none	TWP +ACEI/ARB vs ACEI/ARB	no	24	0.98	0.81,1.16	none	moderate
Liang X.H 2016	8	24/36	425	TWP+CT vs CT	no	90	3.52	0.80,6.25	0.01	moderate
Liao Z.M 2016	16	1～12	1037	TWP+CT vs CT<br>TWP+ACEI/ARB vs ACEI/ARB<br>TWP+CTPM vs CTPM<br>TWP vs ACEI	no	91	2.91	1.05,4.76	0.002	Very low
Dai X.Y 2018	3	NR	NR	TWP +ACEI/ARB vs ACEI/ARB	no	59	6.37	3.54,9.20	< 0.0001	low
Ren D.J 2019	14	1 ~ 6	NR	TWP (+ACEI/ARB) vs ACEI/ARB	total effect	47	4.14	3.43,4.85	NR	moderate
	3		132		follow-up period < 3months	0	1.93	0.80,3.06	0.0008	
	7		377		3months≤follow-up period < 6months	0	3.69	2.92,4.46	< 0.0001	
	9		553		follow-up period≥6months	1	5.20	4.41,6.00	< 0.0001	
Ye W.C 2019	8	1 ~ 6	417	TWP+ valsartan vs valsartan	no	43	3.87	3.12,4.62	< 0.0001	moderate
Wang Y 2020	13	1 ~ 12	817	TWP+ARB vs ARB	total effect	63	3.53	2.44,4.62	< 0.0001	moderate
	5		273		follow-up period < 6months	0	2.53	1.44,3.62	< 0.0001	
	8		544		follow-up period≥6months	72	4.00	2.47,5.53	< 0.0001	
Wu X 2020	12	0.5 ~ 12	727	TWP+ARB vs ARB	total effect	62	5.70	4.44,6.96	< 0.00001	low
	8		445		combined with valsartan	0	4.55	3.65,5.46	< 0.00001	
	4		292		combined with irbesartan	0	8.41	7.14,6.96	< 0.00001	

Zhang M.J 2020	13	2 ~ 6	767	TWP vs CT<br>TWO + ARB vs ARB<br>TWP vs ARB<br>TWP + CTPM vs CTPM	no	67	0.61	0.34,0.87	< 0.0001	Very low

Notes: NR=Not Reported (there is no information provided in the full text version of the included article); NA=Not Applicable; ACEI/ARB = angiotensin-converting enzyme inhibitor/angiotensin II receptor blockade; CTPM=Chinese Traditional Patent Medicine; Ctrl=control; UTP =24hour urinary protein.
