# Supplementary material for: Efficacy and safety of Tripterygium wilfordii polyglycosides for diabetic kidney disease: an overview of systematic reviews and meta-analyses
Source: Syst Rev. 2022 Oct 21;11:226. doi: 10.1186/s13643-022-02091-3 (PMC9585776; doi:10.1186/s13643-022-02091-3)
Supplement: Supplementary file 3 — Additional file 3: Supplemental Table 1. Overview of 24-hour Urinary Protein in the Included SRs and MAs. Supplemental Table 2. Overview of the Included SRs and MAs of Renal Function. Supplemental Table 3. Overview of the Included SRs and MAs about the Outcome of Serum Albumin. Supplemental Table 4. Overview of the Included SRs and MAs of AL. Supplemental Table 5. Overview of the included SRs and MAs about the outcomes of WBC. Supplemental Table 6. Overview of the Incidence of Adverse Events in the Included SRs and MAs. Supplemental Table 7. Methodological Quality Assessment of the Systematic Reviews and Meta-analyses Based on AMSTAR-2 tool. Supplemental Table 8. Quality of Evidence in Included SRs with GRADE. [file 13643_2022_2091_MOESM3_ESM.zip › 13643_2022_2091_MOESM3_ESM/ST-4 ALT 2021-06-21R2.pdf]

Supplemental Table 4. Overview of the Included SRs and MAs of ALT

Study ID (first author, year)	N (studies)	Follow-up period (months)	N (cases)	comparison	subgroups	I^2(%)	MD/SMD (U/L)	95%CI [,]	p	Certainty
Xie HY 2012	10	NR	NR	TWP+Ctrl vs Ctrl	no	NR	3.75	2.98,4.53	< 0.00001	low
Wang Y 2020	7	1 ~ 6	417	TWP+ARB vs ARB	follow-up period	1	1.17	0.54,1.80	0.0003	moderate
Chen H 2020	6	3/6	448	TWP+Ctrl vs Ctrl; TWP+ ACEI/ARB vs Ctrl	no	0	3.73	1.12,12.35	0.03	low
Zhang MJ 2020	14	2 ~ 6	825	TWP vs CT; TWP + ARB vs ARB; TWP vs ARB; TWP + CTPM vs CTPM; comparison with different dose	no	81	0.26	-0.06,0.59	0.11	Very low

Notes: NR: not reported (there is no information provided in the full text version of the included article); ACEI/ARB (angiotensin-converting enzyme inhibitor/angiotensin II receptor blockade); CTPM (Chinese Traditional Patent Medicine); Ctrl(control).
