# Supplementary material for: Efficacy and safety of Tripterygium wilfordii polyglycosides for diabetic kidney disease: an overview of systematic reviews and meta-analyses
Source: Syst Rev. 2022 Oct 21;11:226. doi: 10.1186/s13643-022-02091-3 (PMC9585776; doi:10.1186/s13643-022-02091-3)
Supplement: Supplementary file 3 — Additional file 3: Supplemental Table 1. Overview of 24-hour Urinary Protein in the Included SRs and MAs. Supplemental Table 2. Overview of the Included SRs and MAs of Renal Function. Supplemental Table 3. Overview of the Included SRs and MAs about the Outcome of Serum Albumin. Supplemental Table 4. Overview of the Included SRs and MAs of AL. Supplemental Table 5. Overview of the included SRs and MAs about the outcomes of WBC. Supplemental Table 6. Overview of the Incidence of Adverse Events in the Included SRs and MAs. Supplemental Table 7. Methodological Quality Assessment of the Systematic Reviews and Meta-analyses Based on AMSTAR-2 tool. Supplemental Table 8. Quality of Evidence in Included SRs with GRADE. [file 13643_2022_2091_MOESM3_ESM.zip › 13643_2022_2091_MOESM3_ESM/ST-8R2.pdf]

Supplemental Table 8. Quality of Evidence in Included SRs with GRADE

Included SRs	Outcomes	No of RCTs	Risk of bias	Inconsistency	Indirectness	Imprecision	Others<br>Publication bias	Certainty
Wu W.H. 2010	UTP	11	serious [a]	serious [b]	not serious	not serious	strongly suspected [c]	very low
Xie H.Y. 2012	UTP	22	serious [a]	serious [b]	not serious	not serious	undetected	low
	ALB	15	serious [a]	serious [b]	not serious	not serious	undetected	low
	SCR	21	serious [a]	serious [b]	not serious	not serious	undetected	low
	ALT	10	serious [a]	serious [b]	not serious	not serious	undetected	low
	WBC	5	serious [a]	not serious	not serious	serious [d]	undetected	low
Chen Y 2013	UTP	20	serious [a]	serious [b]	not serious	not serious	strongly suspected [c]	very low
	ALB	11	serious [a]	serious [b]	not serious	not serious	undetected	low
	SCR	16	serious [a]	serious [b]	not serious	serious [d]	undetected	very low
	AEs	17	serious [a]	not serious	not serious	not serious	undetected	moderate
Huang J 2015	UTP	12	serious [a]	serious [b]	not serious	not serious	undetected	low
	ALB	9	serious [a]	not serious	not serious	not serious	undetected	moderate
	SCR	10	serious [a]	not serious	not serious	serious [d]	undetected	low
	AEs	7	serious [a]	not serious	not serious	not serious	undetected	moderate
Lou J.J. 2016	UTP	4	serious [a]	serious [b]	not serious	serious [d]	undetected	very low
	SCR	4	serious [a]	serious [b]	not serious	serious [d]	undetected	very low
Liang X.H. 2016	UTP	6	not serious	serious [b]	not serious	serious [d]	undetected	low
	SCR	7	not serious	serious [b]	not serious	not serious	undetected	moderate
	ALB	8	not serious	serious [b]	not serious	not serious	undetected	moderate
Liao Z.M. 2016	UTP	29	serious [a]	serious [b]	not serious	not serious	strongly suspected [c]	very low
	SCR	24	serious [a]	serious [b]	not serious	not serious	strongly suspected [c]	very low
	ALB	16	serious [a]	serious [b]	not serious	not serious	strongly suspected [c]	very low
	AEs	20	serious [a]	not serious	not serious	not serious	strongly suspected [c]	low
Hong Y 2016	UTP	13	serious [a]	serious [b]	not serious	not serious	undetected	low
	SCR	10	serious [a]	serious [b]	not serious	not serious	undetected	low
	AEs	8	serious [a]	not serious	not serious	not serious	strongly suspected [c]	low
Dia X.Y. 2018	ALB	8	serious [a]	not serious	not serious	serious [d]	undetected	low
	SCR	10	serious [a]	serious [b]	not serious	serious [d]	undetected	very low
	AEs	5	serious [a]	not serious	not serious	serious [d]	undetected	low
Liu K 2019	UTP	16	serious [a]	serious [b]	not serious	not serious	strongly suspected [c]	very low
	SCR	10	serious [a]	serious [b]	not serious	not serious	strongly suspected [c]	very low
	AEs	7	serious [a]	not serious	not serious	not serious	strongly suspected [c]	low
Zhu G.S. 2019	UTP	9	serious [a]	not serious	not serious	not serious	strongly suspected [c]	low
Ren D.J. 2019	UTP	21	serious [a]	serious [b]	not serious	not serious	undetected	low
	ALB	15	serious [a]	not serious	not serious	not serious	undetected	moderate
	AEs	13	serious [a]	not serious	not serious	not serious	undetected	moderate
Ye W.C. 2019	UTP	12	serious [a]	serious [b]	not serious	not serious	undetected	low
	ALB	8	serious [a]	not serious	not serious	not serious	undetected	moderate
	AEs	8	serious [a]	not serious	not serious	not serious	undetected	moderate

Wang Y 2020	UTP	17	serious [a]	not serious	not serious	not serious	strongly suspected [c]	low
	ALB	13	serious [a]	not serious	not serious	not serious	undetected	moderate
	ALT	7	serious [a]	not serious	not serious	not serious	undetected	moderate
	AEs	15	serious [a]	not serious	not serious	not serious	strongly suspected [c]	low
Chen H 2020	ALT	6	serious [a]	not serious	not serious	not serious	strongly suspected [c]	low
	WBC	6	serious [a]	not serious	not serious	not serious	strongly suspected [c]	low
Liu F 2020	UTP	16						very low [f]
	RF	2						very low [f]
	AEs	14						low [f]
Zhang M.J. 2020	UTP	16	serious [a]	not serious	not serious	not serious	strongly suspected [c]	low
	SCR	15	serious [a]	serious [b]	not serious	not serious	strongly suspected [c]	very low
	ALB	13	serious [a]	serious [b]	not serious	not serious	strongly suspected [c]	very low
	ALT	14	serious [a]	serious [b]	not serious	serious [d]	strongly suspected [c]	very low
	AEs	13	serious [a]	serious [b]	not serious	not serious	strongly suspected [c]	low
Fang J.Y. 2020	UTP	9	serious [a]	not serious	not serious	not serious	undetected	moderate
	SCR	8	serious [a]	not serious	not serious	not serious	undetected	moderate
Wu X 2020	UTP	18	serious [a]	serious [b]	not serious	not serious	strongly suspected [c]	very low
	SCR	12	serious [a]	not serious	not serious	not serious	strongly suspected [c]	low
	ALB	18	serious [a]	serious [b]	not serious	not serious	strongly suspected [c]	very low

Explanations

a. Unclear risk of bias for allocation concealment and blinding in most of the included trials, downgrade one level.

b. Statistical significance of heterogeneity (which is serious in all subgroups or unexplained) (downgrade by 1 level)

c. Publication bias is strongly suspected (downgrade by 1 level)

d. Limitations in imprecision with few studies, (few events), downgrade for imprecision.

e. Limitations in imprecision with wide 95% confidence intervals crossing the line of no effect, downgrade for imprecision.

f. We used the assessment from the included SRs

The quality of evidence for each main finding in the comparison was ranked as grades of evidence

'High quality': Further research is very unlikely to change our confidence in the estimate of effect

'Moderate quality': Further research is likely to have an important impact on our confidence in the estimate of effect and may change the estimate

'Low quality': Further research is very likely to have an important impact on our confidence in the estimate of effect and is likely to change the estimate

'Very low quality': We are very uncertain about the estimates
