# Supplementary material for: Efficacy and safety of Tripterygium wilfordii polyglycosides for diabetic kidney disease: an overview of systematic reviews and meta-analyses
Source: Syst Rev. 2022 Oct 21;11:226. doi: 10.1186/s13643-022-02091-3 (PMC9585776; doi:10.1186/s13643-022-02091-3)
Supplement: Supplementary file 3 — Additional file 3: Supplemental Table 1. Overview of 24-hour Urinary Protein in the Included SRs and MAs. Supplemental Table 2. Overview of the Included SRs and MAs of Renal Function. Supplemental Table 3. Overview of the Included SRs and MAs about the Outcome of Serum Albumin. Supplemental Table 4. Overview of the Included SRs and MAs of AL. Supplemental Table 5. Overview of the included SRs and MAs about the outcomes of WBC. Supplemental Table 6. Overview of the Incidence of Adverse Events in the Included SRs and MAs. Supplemental Table 7. Methodological Quality Assessment of the Systematic Reviews and Meta-analyses Based on AMSTAR-2 tool. Supplemental Table 8. Quality of Evidence in Included SRs with GRADE. [file 13643_2022_2091_MOESM3_ESM.zip › 13643_2022_2091_MOESM3_ESM/Supplemental Table 5 [revision_2022-06-05]R2.pdf]

Supplemental table 5. Overview of the included SRs and MAs about the outcomes of WBC

| Study ID (first author, year) | N (studies) | Follow-up period (months) | N (cases) | comparison | subgroups | $I^2$(%) | WBC (10^9/L) | 95%CI [ , ] | p | certainty |
|---|---|---|---|---|---|---|---|---|---|---|
| Xie HY 2012 | 5 | NR | NR | TWP+Ctrl vs Ctrl | no | NR | -0.22 | -0.34,-0.009 | 0.0005 | low |
| Wang Y 2020 | 6 | 1 ~ 6 | 367 | TWP+ARB vs ARB | follow-up period | 0 | -0.19 | -0.51,0.13 | 0.24 | low |
| Chen H 2020 | 6 | 3/6 | 575 | TWP+Ctrl vs Ctrl; TWP+ ACEI/ARB vs Ctrl | no | 0 | OR=4.33★ | 1.08,17.47 | 0.04 | low |
| Zhang MJ 2020 | 10 | 2 ~ 6 | 614 | TWP vs CT; TWP + ARB vs ARB; TWP vs ARB; TWP + CTPM vs CTPM; comparison with different dose | no | 72 | -0.29 | -0.72,0.14 | 0.18 | Very low |

Notes: NR=Not Reported (there is no information provided in the full text version of the included article); NA=Not Applicable; ACEI/ARB =angiotensin-converting enzyme inhibitor/angiotensin II receptor blockade; CTPM=Chinese Traditional Patent Medicine; Ctrl=control. ★OR=Odds Ratio, the authors compared the events of WBC reduced happened in the experimental group to those in the control group.
