# Supplementary material for: Efficacy and safety of Tripterygium wilfordii polyglycosides for diabetic kidney disease: an overview of systematic reviews and meta-analyses
Source: Syst Rev. 2022 Oct 21;11:226. doi: 10.1186/s13643-022-02091-3 (PMC9585776; doi:10.1186/s13643-022-02091-3)
Supplement: Supplementary file 3 — Additional file 3: Supplemental Table 1. Overview of 24-hour Urinary Protein in the Included SRs and MAs. Supplemental Table 2. Overview of the Included SRs and MAs of Renal Function. Supplemental Table 3. Overview of the Included SRs and MAs about the Outcome of Serum Albumin. Supplemental Table 4. Overview of the Included SRs and MAs of AL. Supplemental Table 5. Overview of the included SRs and MAs about the outcomes of WBC. Supplemental Table 6. Overview of the Incidence of Adverse Events in the Included SRs and MAs. Supplemental Table 7. Methodological Quality Assessment of the Systematic Reviews and Meta-analyses Based on AMSTAR-2 tool. Supplemental Table 8. Quality of Evidence in Included SRs with GRADE. [file 13643_2022_2091_MOESM3_ESM.zip › 13643_2022_2091_MOESM3_ESM/Supplemental Table 6 [revision_2022-06-05]R2.pdf]

Supplemental Table 6. Overview of the Incidence of Adverse Events in the Included SRs and MAs

Study ID	N (studies)	follow-up (months)	N (cases)	comparison	subgroups	I^2(%)	OR/RR/RD	95%CI [,]	P	certainty
Chen Y 2013	17	NR	1102	TWP+CT vs CT; TWP +ACEI/ARB vs ACEI/ARB; TWP+CTPM vs CTPM	no	0	OR=4.23	2.42,7.39	< 0.00001	moderate
Huang J 2015	7	NR	406	TWP +ACEI/ARB vs ACEI/ARB	no	0	RD=0.07	0.03,0.12	0.0008	moderate
Hong Y 2016	8	1 ~ 6	NR	TWP +ACEI/ARB vs ACEI/ARB	no	0	OR=6.42	2.23,18.48	< 0.05	low
Liao Z.M 2016	20	1 ~ 12	1526	TWP+CT vs CT; TWP+ACEI/ARB vs ACEI/ARB; TWP+CTPM vs CTPM; TWP vs ACEI	no	0	OR=3.01	1.94,4.67	< 0.00001	low
Dai X.Y 2018	5	NR	NR	NR	no	0	OR=1.23	0.58,2.59	NR	low
Ren D.J 2019	14	1 ~ 6	875	TWP(+ACEI/ARB) vs ACEI/ARB	no	0	RD=0.08	0.05,0.11	< 0.00001	moderate
Ye W.C 2019	8	1 ~ 6	448	TWP+ valsartan vs valsartan	no	0	RR=3.41	1.34,8.66	0.010	moderate
Liu K 2019	7	2 ~ 12	617	TWP+ACEI/ARB vs ACEI/ARB	no	0	RD=0.07	0.03,0.11	0.0002	low
Zhu G.S 2019	9	1 ~ 6	610	TWP vs ACEI/ARB	no	0	OR=1.01	0.56,1.82	0.98	low
Wang Y 2020	15	1 ~ 12	951	TWP+ARB vs ARB	total effect	8	RR=2.22	1.32,3.73	0.003	Low
	6		266		follow-up period < 6months	0	RR=3.90	1.23,12.40	0.02	
	9		685		follow-up period ≥ 6months	37	RR=2.46	1.12,5.41	0.03	
Zhang M.J 2020	13	2 ~ 6	830	TWP vs CT; TWO + ARB vs ARB; TWP + CTPM vs CTPM; comparison with different dose	no	0	RR=1.97	1.22,3.19	0.006	Low
Chen H 2020▲	6	3/6	448	TWP+CT vs CT; TWP+CTPM vs CTPM	no	12	RD=0	-0.04,0.04	0.94	Very low
Liu F 2020	14	2 ~ 6	1014	TWP+CT vs CT (2); TWP+ARB vs ARB (5); TWP+ACEI vs ACEI (2); TWP+ACEI/ARB vs ACEI/ARB (1); TWP vs other agents (1); TWP+ other agents vs other agents (3)	no	0	RR=2.33	1.35,4.02	0.002	Low

Notes: NR: not reported (there is no information provided in the full text version of the included article); NA=not applicable; ACEI/ARB (angiotensin-converting enzyme inhibitor/angiotensin II receptor blockade); CTPM (Chinese Traditional Patent Medicine); Ctrl(control). RR=risk ratio; OR=odds ratio; RD=risk difference. ▲The adverse events were assessed as the incidence of abnormal digestive symptoms, while the incidences of the abnormal level of WBC and ALT were assessed separately.
